# Beyond Expected Patterns in Insulin Needs of People With Type 1 Diabetes: Temporal Analysis of Automated Insulin Delivery Data

**DOI:** 10.2196/44384

**Published:** 2024-11-27

**Authors:** Isabella Degen, Kate Robson Brown, Henry W J Reeve, Zahraa S Abdallah

**Affiliations:** 1 Interactive Artificial Intelligence Centre for Doctoral Training, School of Computer Science, Faculty of Science and Engineering, University of Bristol, 1 Cathedral Square, College Green, Bristol, BS1 5DD, United Kingdom, 44 7726100905; 2 University College Dublin President's Office, College of Engineering and Architecture, University College Dublin, Dublin, Ireland; 3 School of Mathematics, Faculty of Science and Engineering, University of Bristol, Bristol, United Kingdom; 4 School of Engineering Mathematics and Technology, Faculty of Science and Engineering, University of Bristol, Bristol, United Kingdom

**Keywords:** multivariate time series, k-means, clustering, machine learning, temporal patterns, data-driven, OpenAPS, open dataset, type 1 diabetes, insulin needs

## Abstract

**Background:**

Type 1 diabetes (T1D) is a chronic condition in which the body produces too little insulin, a hormone needed to regulate blood glucose. Various factors such as carbohydrates, exercise, and hormones impact insulin needs. Beyond carbohydrates, most factors remain underexplored. Regulating insulin is a complex control task that can go wrong and cause blood glucose levels to fall outside a range that protects people from adverse health effects. Automated insulin delivery (AID) has been shown to maintain blood glucose levels within a narrow range. Beyond clinical outcomes, data from AID systems are little researched; such systems can provide data-driven insights to improve the understanding and treatment of T1D.

**Objective:**

The aim is to discover unexpected temporal patterns in insulin needs and to analyze how frequently these occur. Unexpected patterns are situations where increased insulin does not result in lower glucose or where increased carbohydrate intake does not raise glucose levels. Such situations suggest that factors beyond carbohydrates influence insulin needs.

**Methods:**

We analyzed time series data on insulin on board (IOB), carbohydrates on board (COB), and interstitial glucose (IG) from 29 participants using the OpenAPS AID system. Pattern frequency in hours, days (grouped via k-means clustering), weekdays, and months were determined by comparing the 95% CI of the mean differences between temporal units. Associations between pattern frequency and demographic variables were examined. Significant differences in IOB, COB, and IG across temporal dichotomies were assessed using Mann-Whitney *U* tests. Effect sizes and Euclidean distances between variables were calculated. Finally, the forecastability of IOB, COB, and IG for the clustered days was analyzed using Granger causality.

**Results:**

On average, 13.5 participants had unexpected patterns and 9.9 had expected patterns. The patterns were more pronounced (*d*>0.94) when comparing hours of the day and similar days than when comparing days of the week or months (0.3<*d*<0.52). Notably, 11 participants exhibited a higher IG overnight despite concurrently higher IOB (10/11). Additionally, 17 participants experienced an increase in IG after COB decreased after meals. The significant associations between pattern frequency and demographics were moderate (0.31≤*τ*≤0.48). Between clusters, mean IOB (*P*=.03, *d*=0.7) and IG (*P*=.02, *d*=0.67) differed significantly, but COB did not (*P*=.08, *d*=0.55). IOB and IG were most similar (mean distance 5.08, SD 2.25), while COB and IG were most different (mean distance 11.43, SD 2.6), suggesting that AID attempts to counteract both observed and unobserved factors that impact IG.

**Conclusions:**

Our study shows that unexpected patterns in the insulin needs of people with T1D are as common as expected patterns. Unexpected patterns cannot be explained by carbohydrates alone. Our results highlight the complexity of glucose regulation and emphasize the need for personalized treatment approaches. Further research is needed to identify and quantify the factors that cause these patterns.

## Introduction

Type 1 diabetes (T1D) is a chronic condition where the body produces little or no insulin, a hormone required to regulate blood glucose levels. The principal treatment for T1D is exogenous insulin [1]. Insulin must be skillfully matched to carbohydrate intake to avoid increased blood glucose levels. Beyond carbohydrates, various factors such as exercise, stress, illness, and hormones affect insulin needs [1]. These factors have varying lagging and long-lasting effects and remain underexplored. Hence, insulin dosing remains a complex control task that can go wrong and result in blood glucose levels outside the range that protects people with T1D from adverse health effects [2].

Automated insulin delivery (AID) systems, comprising an insulin pump, a continuous glucose monitor (CGM), and a decision algorithm, represent state-of-the-art T1D treatment [3]. Both commercial [4] and open-source AID systems [5] are becoming more widely adopted. Machine learning research for managing T1D [6] focuses on the safety of AID systems, improving the insulin dosing decision algorithms, improving blood glucose prediction [7,8], predicting hypoglycemia [9], and predicting insulin sets [10] and blood glucose sensor failures [11]. This research uses diverse machine learning methods including support vector machines [12], random forests [13], and combined approaches [14]. In [13], random forests were used to predict blood glucose levels, leveraging multivariate data on daily rhythms in glucose metabolism. Data used for these studies were either from simulated patients [15] or collected in clinical settings, including around 5‐30 people. Crucially, research efforts concentrate primarily on predicting blood glucose levels to inform the more ambitious task of controlling these levels, which requires knowledge of the causal structure. Our research uses a data-centric approach to explore the effects of lesser-known causal interactions in glucose metabolism.

Both commercial and open-source AID solutions effectively regulate blood glucose levels [16-22]. Although AID data are often used to assess clinical outcomes and system safety, its potential for broader research remains largely untapped. One study that goes beyond clinical outcomes has looked at blood glucose outcomes and variability concerning gender [23]. Other T1D research focuses on issues such as predicting diabetes onset [24], predicting changes in behavior, and evaluating the efficacy of treatment. These researchers stress the importance of data-driven methods and the shift toward tailored management of therapies. Research into AID data offers insights into glucose regulation in free-living conditions. Further advantages of AID data include more comprehensive and accurate datasets than manual treatment records, as well as more consistent, indefatigable, undistracted, emotionless, and replicable insulin dosing decision-making by an algorithm compared to human decision-making. However, open-source AID data collected in real-life conditions come with the challenges of irregularities, noneven sampling, and missing data, which make it hard to handle the data with current time series techniques. In this study, we use the OpenAPS Data Commons dataset, which is an extensive dataset collected in real-life conditions from 183 people with T1D who use an open-source AID system [25]. From the AID device’s extensive system logs, we are focusing on the insulin on board (IOB), carbohydrates on board (COB), and interstitial glucose (IG) information. The insulin and carbohydrates “on board” values are calculations of the AID to model how much insulin and carbohydrates are active at any point in time [26,27].

The goal of insulin dosing is euglycemia, the state when blood glucose levels are within the normal range. In clinical practice, carbohydrate intake is considered the most important factor in determining insulin needs [2]. Insulin needs are estimated by monitoring glucose levels after carbohydrate intake or fasting. If glucose levels remain in the normal range, the insulin needs for that time are met; if glucose levels rise, the insulin dose is too small or late; and if glucose levels drop, the insulin dose is too high or early [2,28-30]. This experimentation has been formalized—for example, in the educational program called Dose Adjustment for Normal Eating—and been shown to improve outcomes but with mixed long-term success [31]. We know that euglycemia is the result of a complex interplay of metabolic processes that lower and increase blood glucose levels and change the effect that hormones like insulin have [32-34]. Unobserved confounding factors influencing insulin needs include other macronutrients, exercise, stress, and menstrual cycle. Fat and protein affect glucose levels by impacting carbohydrate absorption and being broken down into glucose [35]. Exercise triggers a complex neuroendocrine response that is impaired in people with T1D and necessitates insulin adjustment [36]. Stress alters glucose metabolism and insulin production [37,38], while the fluctuation in female hormones continuously changes insulin requirements [39]. These factors lead to unexpected situations, for example, when eating carbohydrates does not lead to increased glucose levels, and glucose levels increasing in the absence of carbohydrate intake. Currently, these factors are not continuously measured and not systematically considered in insulin dosing [1,2,30].

This study aims to identify and quantify expected and unexpected temporal patterns in insulin needs using AID data. AID data provides a novel opportunity to research the impact of unobserved confounding factors on blood glucose due to the automatic (albeit with a lag) insulin dose adjustment by the algorithm attempting to keep IG within a specified range. Our hypothesis is that unexpected temporal patterns in insulin needs are common in AID data. Our findings aim to encourage more research into less-explored factors that change insulin needs and to use this information to improve insulin dosing decision-making. We further hope to motivate more research into time series pattern-finding methods that can deal with this complex type of real-life system data.

## Methods

### Overview

The methods and results are organized as follows: we describe the data and participants; explain how similar days were grouped; define the “expected” and “unexpected” patterns; analyze how common the patterns are for various time resolutions; investigate the relationship between pattern frequency and demographic factors; explore how IOB, COB, and IG compare across various temporal dichotomies; and finally study whether past values of IOB, COB, and IG can predict each other.

### Data and Population

**Figure 1.**
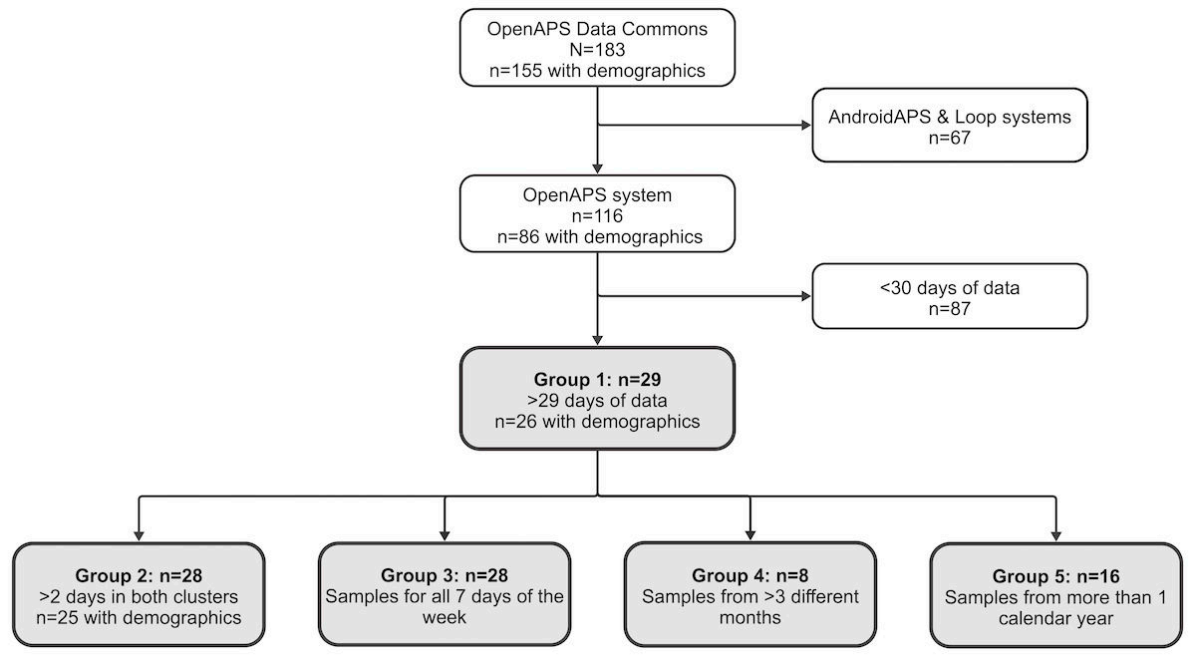
Flowchart of the selection process for the 29 people whose data were used in this study. Groups 2‐5 are subgroups of the participants from group 1 with sufficient data for the different time resolutions.

We analyzed the OpenAPS Data Commons dataset, which consists of open-source AID data collected in free-living conditions from people with T1D and their self-reported demographic data [25]. OpenAPS was selected (n=116), being the most frequently used system, excluding the AndroidAPS and Loop systems. From the OpenAPS device status files, the enacted time stamp and IOB, COB, and IG (called BG in the dataset) values were read and processed into regularly, hourly sampled and equal length daily segments for each person. The time stamps were made uniform by translating the different formats into UTC. Time stamps without time zone information were imputed with time zone information from previous time stamps and missing time stamp entries were dropped. The irregular time series were resampled into regular time series with an hourly frequency aggregating values into a mean value. To avoid resampling over periods without sufficient data points, days with less than 1 reading per hour were dropped. Note that this excludes days when the system was interrupted for more than an hour, such as when the CGM sensor was changed. To ensure we gained a representative picture of patterns for each participant, we excluded people with less than 30 days of data [40]. The code to preprocess the OpenAPS Data Commons data into regularly, hourly sampled time series has been made available [41]. The population whose patterns were analyzed in detail is group 1 (n=29). Demographic information was available for 26 of the individuals. Figure 1 shows how the people were selected. Group 1 consists of the participants who have more than 29 days of data; group 2 (n=28) is the subgroup of them who have at least 3 days in each cluster; group 3 (n=28) is the subgroup with data from all 7 days of the week; group 4 (n=8) is the subgroup with data from at least 4 different months that include December, January, February, June, July, and August; and group 5 (n=16) is the subgroup with data from more than 1 year.

To describe the resulting data, we calculated the mean, SD, range, and amount of data for IOB, COB, and IG, as well as the demographic data for group 1. For individuals with multiple demographic reports, we used the report closest to the most current AID record. For IOB, COB, and IG, we investigated the distribution properties including kurtosis, skew, number of modes, and whether IOB, COB, and IG follow a normal distribution. We used Python for all analyses. To calculate the distribution properties, we used Pandas [42] and NumPy [43], while to test for normal distribution, we used SciPy [44].

### Clustering Similar Days

To group similar days, we used time series k-means clustering. To prevent bias from different measurement scales, we applied min-max scaling of IOB, COB, and IG values to a range of 0 to 10 for each participant. Min-max scaling does not change the distribution, but it ensures that IOB (measured in U) with a value range in the low tens, COB (measured in grams) with a value range in the tens, and IG (measured in mg/dL) with a value range in the low hundreds all have the same importance for the distance calculation [45,46]. K-means clustering requires specifying the number of clusters k. We determined k using silhouette analysis [47,48]. Silhouette analysis calculates the distance between all days and compares the average distance of days in the same cluster to those in other clusters. The resulting average silhouette score is a number between −1 and 1. Higher silhouette scores indicate that the days in each cluster are similar and that the clusters are well separated. Negative scores convey that the days in a cluster are closer to days in other clusters. Both silhouette analysis and k-means clustering require an appropriate similarity measure to compare the time series. We evaluated Euclidean, dynamic time warping (DTW) [49], and SoftDTW [50] distances. The Euclidean distance is calculated by adding the difference between each hour of the day and dividing the total by 24. DTW and SoftDTW attempt to align similar elements between the time series by allowing the hours compared to warp. Despite DTW resulting in higher average silhouette scores, we used the Euclidean distance, as it allows us to compare the same hours of the day between the clusters. The optimal number of clusters for most participants was 2, which we used for everyone. We presented the time series clustering used in this study in more detail at the NeurIPS 2022 Time Series for Health Workshop [51]. For clustering, we used TSLearn [52]; for scaling, we used scikit-learn [53].

### Pattern Definition

We defined the “expected” and “unexpected” patterns based on the logic of the standard trivariate model for IOB, COB, and IG typically used for insulin dosing, as shown in Table 1. The patterns are determined by sequentially setting the level of each of the 3 variables higher than usual and then assigning the expected or unexpected level of the variable considered causal to the higher levels observed in the first variable. This results in 3 “expected” patterns of insulin need for which the trivariate model works (E1-E3) and 3 “unexpected” patterns of insulin need in which unobserved confounders override the logic of the standard model (U1-U3). Expected pattern E1 describes situations when IOB is higher than usual, therefore COB is expected to be higher than usual, and IG is similar (IOB matches COB well), lower (too much IOB), or higher (too little IOB). In the unexpected form of this pattern U1, when IOB is higher, COB unexpectedly is similar or lower, and IG unexpectedly is similar (IOB matches confounders well) or higher (too little IOB to cover confounders). Expected pattern E2 describes situations when IG is higher than usual, therefore COB is expected to be higher than usual, and IOB is similar (too little IOB to cover more COB), lower (IOB mistakenly reduced), or higher (IOB not sufficiently increased). In the unexpected form of this pattern U2, when IG is higher, COB unexpectedly is similar or lower, and IOB unexpectedly is similar (too little IOB for confounders) or higher (IOB not sufficiently increased to cover confounders). Finally, expected pattern E3 describes the same situations as pattern E1 but the causal variable for higher COB this time is IOB. Therefore, in the unexpected form of this pattern U3, when COB is higher than usual, IOB unexpectedly is similar or lower, and IG unexpectedly is similar (COB matches confounders) or lower (too little COB to cover confounders).

**Table 1.** Overview of expected (E1-E3) and unexpected (U1-U3) patterns of insulin needs in type 1 diabetes using the standard trivariate insulin on board (IOB), carbohydrates on board (COB), and interstitial glucose (IG) insulin dosing model. In each of the 3 expected and 3 unexpected patterns, we observed a significantly higher mean level than usual in 1 of the 3 variates for a specific time unit.^a^ The mean observed level of the variate thought to be causing this change is marked with ^b^ and the mean observed level for the third variate with ^c^. If the level of the causal variate and the third variate follow the model, it is an expected pattern; if not, it is an unexpected pattern.

Pattern description			Observed levels for variates		
			IOB	COB	IG
**Expected patterns of insulin needs (trivariate model works)**					
	E1	Higher IOB is needed for higher COB	Higher^a^	Higher^b^	Any^c^
	E2	Higher IG is due to higher COB	Any^c^	Higher^b^	Higher^a^
	E3	Higher COB needs higher IOB	Higher^b^	Higher^a^	Any^c^
**Unexpected patterns of insulin needs (confounding factors involved)**					
	U1	Higher IOB is not due to higher COB	Higher^a^	Similar, lower^b^	Similar, higher^c^
	U2	Higher IG is not due to higher COB	Similar, higher^c^	Similar, lower^b^	Higher^a^
	U3	Higher COB does not require higher IOB	Similar, lower^b^	Higher^a^	Similar, lower^c^

a Variate for which a significantly higher mean level than usual is observed for a specific time unit.

b Expected/unexpected mean level for the variate thought to be causing the difference in variate.

c Expected/unexpected mean level observed for the leftover third variate.

### Frequency of Expected and Unexpected Patterns

To determine how often patterns occur, we counted the frequency of all patterns defined in Table 1 for each participant by calculating the 95% CI of the differences in means between the different time units. We counted the patterns for the following time resolutions: comparing the hours of the day, the same hours of the day between the 2 clusters, the days of the week, and the months of the year. For each comparison, the means μ_1_ and μ_2_ were deemed similar when 0 was part of the CI, lower when CI<0 (μ_1_<μ_2_), and higher when CI>0 (μ_1_>μ_2_). We applied a Bonferroni correction to control the family-wise error rate at 5% for each time resolution. Therefore, for hours of the day, α was adjusted to .0002; for the clusters, α was adjusted to .0021; for days of the week, α was adjusted to .0024; and for months of the year, α was adjusted to .0008. Participants in group 1 were used for hours of the day, group 2 for clusters, group 3 for days of the week, and group 1 for months of the year (see Figure 1 for a description of the groups). We calculated the empirical effect size *d* = (μ_1_ – μ_2_) / s_p_ (Equation 1) using the pooled standard deviation s_p_ = square root(((n_1_ – 1) s_1_^2^ + (n_2_ – 1) s_2_^2^) / (n_1_ + n_2_ – 2)) (Equation 2) [54]. Note that n_1_ and n_2_ are the numbers of observations, and s_1_ and s_2_ are the standard deviations of the time units compared. Further, we calculated the number of observations n that would be required for a power of 80% using n = 2((z_α/2_ + 0.84) / *d*)^2^ (Equation 3) [54], where z_α/2_ is the critical value of the Bonferroni adjusted α for each time resolution.

### Relationships Between Pattern Frequency and Factors

Kendall τ [55] variant b (to allow for ties [56]) was used to assess the relationships between pattern frequency and the self-reported demographics for the different participants, except for sex, for which we only had 2 females and 7 males. We selected Kendall τ as it can relate continuous and ordinal variables and deals well with outliers [57]. Further, unlike Pearson *r*, it can handle skewed variables [57] and assess nonlinear relationships. Compared to Spearman ρ, Kendall τ provides more protection against type I errors in severe conditions, requires smaller sample sizes, and is easy to interpret [57]. We calculated both the strength of the relationship τ and the statistical significance *P*. The null hypothesis stated that the 2 variables are not related (*τ*=0) using the 2-tailed alternative hypothesis. Depending on the presence of ties, *P* is calculated using either the exact or asymptotic method [56]. We calculated Kendall τ using SciPy [44], which offers an automatic option for the *P* calculation method. For relationships where *P*<.05, we concluded that the relationship was statistically significant. Further, we calculated the number of participants that would be needed for a power of 80% as n = 4+0.437((z_α/2_ + z_β_) / (z(τ_b1_) – z(τ_bo_)))^2^ (Equation 4) [58], where z_α/2_=1.96, z_β_=0.84, z(τ_b1_) is the Fisher *z* transformed value of τ, and z(τ_b0_) is the Fisher *z* transformed value for the null hypothesis *τ*=0, which is 0.

### Comparison of IOB, COB, and IG

We compared IOB, COB, and IG across the following 4 temporal dichotomies: the clusters of similar days; workdays (Monday-Friday) versus weekends (Saturday and Sunday); winter (December-February) versus summer months (June-August); and first versus second year of AID data. This comparison was performed using the nonparametric 2-tailed Mann-Whitney *U* test [59], where we rejected the null hypothesis that μ_1_=μ_2_ when *P*<.05. The empirical effect size *d* was calculated using Equation 1 but with the simple pooled standard deviation s_p_ = square root((s_1_^2^ + s_2_^2^) / 2). The number of participants required for a power of 80% for each temporal category was established using the empirical effect sizes and *α*=.05. We then grouped the participants with significant opposite effects by comparing the 95% CIs of mean differences. For this comparison, we applied a Bonferroni correction to control the family-wise error rate at 5%. Therefore, to compare the 2 clusters and the weekdays and weekends, α was adjusted to .0018; to compare winter and summer months, α was .0063; and to compare the first and the second year, α was .0031. The effect size *d* was calculated using Equation 1. Finally, to further compare the means between the 2 clusters, we visually inspected the clusters’ 95% CIs of mean IOB, COB, and IG, and calculated the Euclidean distance between these means. We used group 2 to compare clusters, group 3 to compare workdays and weekends, group 4 to compare winter and summer months, and group 5 to compare the first and second year of AID data. For the 3 people who had days from 3 different years in group 5, we used the 2 years with more data. Given that k-means clustering randomly assigned the cluster labels for each participant, we consistently labeled the clusters, calling the cluster with the lower mean IOB “cluster 1.” For the Mann-Whitney *U* tests, we used SciPy [44]; for the power calculation, we used G*Power software (version 3.1.9.6; Erdfelder, Buchner, and Lang [60]).

### Forecastability of IOB, COB, and IG

We explored whether IOB, COB, and IG can forecast each other for each cluster assessing Granger causality. Granger causality between 2 time series determines if 1 time series carries information about the other time series to forecast it at a certain lag (delay) [61,62]. We investigated a lag of 1 hour, 2 hours, and 3 hours. We concluded that the lag that achieves the lowest *P* value for the Granger causality works the best. The time series investigated need to be stationary. It is common practice to run an augmented Dickey-Fuller (ADF) unit root test to test for the stationarity of a time series [63,64]. If the mean of a cluster was not stationary, we took the derivative until we passed the ADF test for stationarity with *P*<.05 for all variates. Note that in Granger causality, if variate 1 can be used to forecast variate 2, the opposite is not necessarily true. Therefore, we evaluated Granger causality for all pairwise permutations of IOB, COB, and IG. Statmodels [65] was used to calculate Granger causality and for the ADF test. This method runs 4 different statistical tests, and we required all of them to have *P*<.05 to infer Granger causality. We calculated the derivative using NumPy [43].

### Ethical Considerations

The Faculty of Engineering Research Ethics Committee of the University of Bristol reviewed and approved this study. The ethics approval code is 11270. This ethics application included and extended the OpenAPS Data Commons research guidelines for working with the OpenAPS Data Commons dataset as set out by the community, which can be found on their website [66]. The application also included the permission granted to research this data by the OpenAPS Data Commons administrator. The OpenAPS Data Commons data and the demographic data used in this study have been voluntarily donated via the Open Humans platform by people who use an open-source AID or their parents. The Open Humans platform guides the participants through the donation process and automatically deidentifies them using a numerical ID that must not be published. Participants can recall their data at any time, which forces us to delete their data from our copy of the data. The uploading of data is ongoing; we worked with a version of the dataset from April 2022. We have further improved the participants’ privacy by excluding their free-form notes sometimes present in the data and using UTC timestamps without geolocation information. The participants have not received compensation for donating their data.

## Results

### Data and Population

A total of 29 participants had at least 30 days of data with at least 1 reading each hour. Table 2 shows the number of participants (n), the mean (SD), and the range of values for all the characteristics. In addition, 21 of the 29 participants reported having T1D themselves, 5 are parents of a child with T1D, and we did not have this information for the remaining 3 participants. For 19 participants, sex was unknown; there were 7 reported to be male and 3 reported to be female.

**Table 2.** Characteristics from automated insulin delivery device and accompanying demographic data for group 1 (n=29).

Characteristic		Participants, n	Mean (SD)	Range
**Automated insulin delivery device data^a^**				
	Hours (count)	29	1923.3 (1807.9)	720‐9024
	Days (count)	29	80.14 (75.3)	30‐376
	Months (count)	29	6.38 (3.7)	3‐19
	Years (count)	29	1.62 (0.6)	1‐3
	Most recent year recorded (year)	29	2018.66 (1.2)	2017‐2021
	Insulin on board (U)	29	1.66 (0.9)	0.41‐3.67
	Carbohydrates on board (grams)	29	9.84 (5.3)	1.73‐24.08
	Interstitial glucose (mg/dL)	29	133.92 (16.5)	98.15‐162.45
**Demographics data^b^**				
	Age (years)	25	36.4 (16.6)	8‐66
	Duration with type 1 diabetes (years)	26	21.7 (14.7)	1‐56
	Last lab-reported glycated hemoglobin A_1c_ (mmol/mol)	25	46 (7.9)	34.4‐60.66
	Average carbohydrates (grams/day)	25	156.7 (72.2)	20‐330
	Average insulin (U/day)	23	41.1 (20.5)	14‐89
	Average basal insulin (U/day)	24	22.6 (15.4)	8.6‐69.8
	Pumping since (year)	25	2006.9 (8.8)	1980‐2018
	Continuous glucose monitoring since (year)	25	2013.5 (3.5)	2006‐2018
	Automated insulin delivery since (year)	25	2017.1 (0.9)	2016‐2020
	Demographics reported (year)	26	2017.8 (1)	2017‐2020

a Automatically recorded.

b Self-reported.

From Table 2, we can see that group 1 covers a good range of people regarding age and duration of T1D. The participants had excellent glucose control, with an average lab-reported glycated hemoglobin A_1c_ (HbA_1c_) of 46 mmol/mol (SD 7.9). The National Institute for Health and Care Excellence recommends HbA_1c_≤48 mmol/mol, which in the United Kingdom, 9.8% of people with T1D achieve, and 19.5% achieve <53 mmol/mol [67,68]. All the people lived in Western countries (19 in North America, 6 in Europe, 1 in Oceania, and 3 unknown). Further, the participants were early adopters of diabetes technology. On average, they started pumping in 2006, using a CGM in 2013, and using an AID system in 2017. In 2009, the uptake of insulin pumps in T1D in the USA was 43.2%, while the uptake of CGM was 10.5%; in 2019, insulin pumps were used by 54.4% (this percentage is lower for the United Kingdom) of people with T1D and and 40.9% used CGM [69]. Open-source AID systems were the forerunners of commercially available AID systems, with over 100 users in 2016 [70]. Commercial systems became available around 2017 [4].

On average, the participants reported eating 156.7 grams (SD 72.2) of carbohydrates per day. A total of 10 people reported eating a standard amount of carbohydrates (>130 grams/day), 17 people ate a low-carbohydrate diet (50‐130 grams/day), and 2 people ate a very low carbohydrate diet of <50 grams/day [71]. Only 1 person reported eating more than 266 grams/day. The total amount of daily insulin varied from person to person (mean 41.1 U, SD 20.5).

IOB, COB, and IG did not follow a normal distribution for any participant. We rejected the null hypothesis of the normal test with *P*<.001. The distribution characteristics varied considerably between the individuals. The kurtosis results (range −0.2 to 26.18) indicated that these distributions are more peaked than a normal distribution, with one exception where a single participant showed a flatter IG distribution. The skew results indicated that lower values are more frequent than higher values (range 0.06 to 4.9). Regarding the most frequent value (mode), only COB had a mode of 1 for everyone. IOB and IG were multimodal, IOB (range 1‐22) more so than IG (range 1‐3). A total of 21 people had 1 IG mode, 6 had 2, and 2 had 3. IOB had more variations: only 12 people had 1 mode, and 4 had 3 modes. One person had 22 modes, another had 21; for the others, the number of modes was ≤10 (see Table S1 in Multimedia Appendix 1 for more details).

### Clustering Similar Days

The Euclidean distance achieved the lowest average silhouette scores (mean silhouette score 0.17, SD 0.09) and SoftDTW the highest (mean 0.32, SD 0.016; see Table S2 in Multimedia Appendix 1). Creating 2 clusters achieved the highest silhouette score for 21 of 29 participants in group 1. For the other 8 participants, the best cluster number k varied between 3 and 16, with Euclidean silhouette scores ranging from 0.07 to 0.19 (see Table S3 in Multimedia Appendix 1).

### Frequency of Expected and Unexpected Patterns

Our results found that unexpected patterns were as frequent as their expected counterparts. Table 3 shows the number of participants with 1 or more occurrences of a specific pattern for a given time resolution. The number of participants with patterns varied for the different time resolutions. Averaging across patterns, 21 participants had expected patterns (E1-E3) for hours of the day, while 23 had unexpected patterns (U1-U3). For clusters, 8.67 had expected patterns and 11.7 had unexpected patterns; for the days of the week, 2.67 had expected patterns and 8 had unexpected patterns. For months of the year, 7.33 had expected patterns and 11.3 had unexpected patterns. Averaging across the different time resolutions and patterns, 9.9 participants had expected patterns and 13.5 participants had unexpected patterns. Note that the participants with patterns in one of the time resolutions are not necessarily the same individuals in another. Not all participants had patterns. When summing up all expected and unexpected patterns across all time resolutions, 3 participants had no expected pattern in any of the time resolutions. All participants had at least one unexpected pattern in 1 or more of the time resolutions. However, for hours of the day, 2 participants of 29 had no patterns; for clusters, 6 of 28; for days of the week, 15 of 29; and for months of the year, 10 of 29. For 1‐2 participants, the unexpected patterns continued to appear in the hours of the day and clusters in their most pronounced form for which the means of all 3 variates were forced to be significantly different in contradictory ways. The frequency of patterns varied greatly across the participants. The mean occurrence of the expected patterns was 76.1, SD 67.24, range 0‐265. For unexpected patterns, the mean occurrence was 47.14, SD 31.35, range 6‐127. The difference in occurrence between expected and unexpected patterns overall was not statistically significant (*P*=.08; 1-tailed Mann-Whitney *U* test with the alternative hypothesis that expected patterns are more frequent than unexpected patterns). Comparing the sum of the frequency of the expected forms of a pattern with their unexpected forms showed that expected pattern E1 was more frequent than unexpected pattern U1 (*P*=.046) and U3 (*P*<.001) but expected pattern E2 was less frequent than its unexpected form U2 (*P*=.007), all 1-tailed Mann-Whitney *U* tests. Unexpected pattern U2 was the most common among participants; it describes situations when IG is higher but COB is not (see Table 1 for a description of the patterns). The second most common pattern was U1, which describes situations where IOB is significantly higher but COB is not. The least common pattern was expected pattern E2, which describes situations when COB and IG are significantly higher. The latter indicates that the AID did a good job of reducing the impact of COB on IG. In general, patterns were most common in hours of the day, second most when comparing the same hours of the day between the 2 clusters, third most when comparing months of the year, and least common in days of the week. This suggests that these patterns occur in a circadian and seasonal rhythm, rather than a weekday rhythm. However, these results are also influenced by the amount of data available for each time resolution. U*i*’, U*i*’’, and U*i*’’’ are stricter variations of the original pattern (*i*=1,2,3).

**Table 3.** Number of participants with at least 1 occurrence of the expected or unexpected pattern, comparing hours of the day, the same hour of the day between clusters, days of the week, and months of the year.

Pattern		Observed levels for variates			Number of people with the pattern n				Mean n (SD)
		Insulin on board	Carbohydrates on board	Interstitial glucose	Hours of the day	Clusters	Days of the week	Months of the year	
**Expected patterns of insulin needs**									
	Mean of E1-E3	—^a^	—	—	21	8.7	2.7	7.3	9.9 (7.82)
	E1	Higher^b^	Higher^c^	Any^d^	24	9	3	8	11 (9.06)
	E2	Any^d^	Higher^c^	Higher^b^	15	16	11	14	7.8 (5.44)
	E3	Higher^c^	Higher^b^	Any^d^	24	9	3	8	11 (9.06)
**Unexpected patterns of insulin needs**									
	Mean of U1-U3	—	—	—	23	11.7	8	11.3	13.5 (6.55)
	U1	Higher^b^	Similar, lower^c^	Similar, higher^d^	26	13	9	8	14 (8.29)
	U2	Similar, higher^d^	Similar, lower	Higher^b^	23	16	11	14	16 (5.1)
	U3	Similar, lower^c^	Higher^b^	Similar, lower^d^	20	6	4	12	10.5 (7.19)
**Unexpected patterns, most pronounced form**									
	U1’	Higher^b^	Lower^c^	Higher^d^	1	1	0	0	0.5 (0.58)
	U2’	Higher^d^	Lower^c^	Higher^b^	1	1	0	0	0.5 (0.58)
	U3’	Lower^c^	Higher^b^	Lower^d^	2	0	0	1	0.5 (0.58)
**Unexpected patterns, not allowing similar in third variate^d^**									
	U1’’	Higher^b^	Similar, lower^c^	Higher^d^	19	12	5	4	10 (6.98)
	U2’’	Higher^d^	Similar, lower^c^	Higher^b^	19	12	5	4	10 (6.98)
	U3’’	Similar, lower^c^	Higher^b^	Lower^d^	8	1	0	3	3 (3.56)
**Unexpected patterns, not allowing similar in “causal” variate^c^**									
	U1’’’	Higher^b^	Lower^c^	Similar, higher^d^	1	1	0	1	0.75 (0.5)
	U2’’’	Similar, higher^d^	Lower^c^	Higher^b^	9	3	3	0	3.75 (3.76)
	U3’’’	Lower^c^	Higher^b^	Similar, lower^d^	2	0	0	1	0.75 (0.96)

a Not applicable.

b Variate for which a significantly higher mean level than usual is observed for a specific time unit.

c Expected/unexpected mean level for the variate thought to be causing the difference in variate.

d Expected/unexpected mean level observed for the third variate.

The mean effect sizes ranged from 0.97 to 1.27 for hours of the day and clusters. Smaller mean effect sizes were observed for days of the week and months of the year (0.3<*d*<0.52; see Table 4 for details). The number of observations varied for the 3 variates (IOB, COB, and IG) and the different time resolutions, resulting in a range of n_1_ (number of observations for variate 1) and n_2_ (number of observations for variate 2). Therefore, Table 4 includes the 25% and 50% quantiles of the effect sizes and the number of observations for the variable with fewer observations min(n_1_, n_2_). To achieve a power of 80% for effect sizes ≥0.8, a total of 66 observations for hours of the day would be required, 48 for clusters, 47 for days of the week, and 56 for months of the year. For an effect size of 0.5, we would need 169 observations for hours of the day, 123 for clusters, 121 for days of the week, and 142 for months of the year (see Table S4 in Multimedia Appendix 1 for other effect sizes). We achieved a power of 80% for the effect sizes at 50% quantiles, except for lower IG for clusters and higher COB for days of the week.

**Table 4.** Mean, SD, range, 25% and 50% quantiles of empirical effect size *d*, and 25% and 50% quantiles for minimum number of observations in group 1 (n_1_) or 2 (n_2_) for significantly lower and higher differences in mean insulin on board (IOB), carbohydrates on board (COB), and interstitial glucose (IG) across the different time resolutions.

Time resolution and variates		Lower differences						Higher differences					
		Cohen *d*				min (n_1_,n_2_)		Cohen *d*				min (n_1_,n_2_)	
		Mean (SD)	Range	25%	50%	25%	50%	Mean (SD)	Range	25%	50%	25%	50%
**Hours of the day**													
	IOB	1.27^a^ (0.54)	0.30‐3.65	0.90	1.16^a^	45	67	1.15^a^ (0.51)	0.27‐3.67	0.82	1.07^a^	45	74
	COB	1.12^a^ (0.47)	0.29‐3.47	0.82	1.02^a^	45	67	1.09^a^ (0.45)	0.27‐2.89	0.80	1.00^a^	45	74
	IG	0.97^a^ (0.41)	0.28‐3.15	0.70	0.90^a^	47	92	0.94^a^ (0.36)	0.27‐2.21	0.68	0.88^a^	53	92
**Clusters**													
	IOB	1.41^a^ (0.87)	0.49‐5.59	0.93	1.11^a^	23	27	1.21^a^ (0.66)	0.44‐4.95	0.82	1.03^a^	31	57
	COB	1.53^a^ (1.15)	0.52‐5.51	0.76	1.07^a^	14	28	1.30^a^ (1.36)	0.35‐8.19	0.75	0.92^a^	24	35
	IG	1.26^a^ (0.42)	0.35‐2.21	1.02	1.19	23	26	1.08^a^ (0.44)	0.37‐3.16	0.82	1.02^a^	31	42
**Days of the week**													
	IOB	0.41^a^ (0.24)	0.18‐1.60	0.25	0.38^a^	120	204	0.42^a^ (0.15)	0.13‐0.77	0.29	0.41^a^	102	264
	COB	0.30^a^ (0.13)	0.12‐0.54	0.20	0.26^a^	96	504	0.38 (0.10)	0.23‐0.57	0.36	0.39	24	72
	IG	0.41 (0.16)	0.14‐0.88	0.25	0.46^a^	96	168	0.41 (0.19)	0.14‐0.82	0.27	0.42^a^	144	216
**Months**													
	IOB	0.40^a^ (0.17)	0.16‐0.97	0.28	0.33^a^	264	384	0.52^a^ (0.29)	0.18‐1.97	0.32	0.44^a^	312	384
	COB	0.42^a^ (0.17)	0.25‐0.92	0.29	0.38^a^	144	336	0.38^a^ (0.16)	0.18‐0.92	0.27	0.34^a^	198	384
	IG	0.40^a^ (0.16)	0.17‐1.22	0.29	0.35^a^	288	384	0.43^a^ (0.19)	0.19‐1.22	0.31	0.39^a^	216	336

a Effect sizes with a power of ≥80%.

### Relationships Between Pattern Frequency and Factors

The demographic factors that were significantly associated with the frequency of patterns were the last lab-reported HbA_1c_ (pattern E1 for days of the week, pattern E2 for months of the year, pattern U1 for hours of the day, pattern U2 for months of the year), average insulin (pattern U1 for hours of the day), and pumping since (pattern U2 for clusters and months of the year); see Table 5 for more information. These associations were of medium strength ±0.3<τ<±0.5. Age, duration of T1D, average carbohydrates, average basal insulin, using CGM since, and using AID since were not significantly associated with the frequency of any pattern. Further, mean COB (pattern E1 for hours of the day) and mean IG (pattern E1 for clusters and days of the week, pattern E2 for clusters, pattern U1 for hours of the day), as well as number of hours, days, months, and years were also significantly associated with the frequency of some patterns. Mean IOB was not significantly associated with the frequency of any pattern. The significant associations between pattern frequency and the amount of data were all positive. This shows that the pattern frequency increases with more data (see Table 2 for the mean amount of data). These relationships were not significant for all patterns and time resolutions. The number of factors with a significant association was similar for each pattern except for pattern U3, which was not significantly associated with any factor other than the amount of data (Table 5). Further, we found a significant relationship for the last lab-reported HbA_1c_ with the frequency of pattern E1 (E1 increased as HbA_1c_ decreased for days of the week), the frequency of pattern E2 (E2 increased with HbA_1c_ for patterns in months of the year), and the same for pattern U1 in hours of the day and pattern U2 in months of the year. For self-reported average insulin, the frequency of pattern U1 increased as insulin decreased. Additionally, the frequency of pattern U2 increased when people used a pump less long in clusters and months of the year. Note that “pump since” was provided as a year, therefore higher numbers mean that a pump has been used for a shorter period. The frequency of pattern E1 increased when the AID device recorded higher mean COB in hours of the day. Finally, the lower the mean IG was, the higher the frequency of pattern E1 in clusters and days of the week. The same was noted for the frequency of pattern E1 in clusters. However, the frequency of pattern U1 increased when the mean IG in hours of the day was higher. Overall, pattern U3 had the weakest association with any factors and pattern U2 had the strongest. Patterns E1-E3 and U1 had similarly strong associations with factors. Differences in association strength between the time resolutions were not pronounced. Days of the week had the fewest significant associations and months of the year had the most. The latter, however, was most often associated with the amount of data. Unsurprisingly, patterns in months were only found in participants with more than 1 month of data.

**Table 5.** Kendall τ associations between expected (E1-E3) and unexpected (U1-U3) patterns and factors with significant associations for each time resolution. The results for all factors can be found in Table S5 in Multimedia Appendix 1.

Pattern and time resolution			Last glycated hemoglobin A_1c_	Average insulin	Pumping since	Mean COB^a^	Mean IG^b^	Hours count	Days count	Months count	Years count
Participants for clusters			24	22	24	28	28	28	28	28	28
Participants for others			25	23	25	29	29	29	29	29	29
**E1 (more IOB^c^ is needed for more COB) and E3 (more COB is needed for more IOB)**											
	***Hours of the day***										
		τ	0.13	0	0	0.45^d^^,^^e^	0.11	0.38^d^^,^^e^	0.38^d^^,^^e^	0.18	0.11
		*P* value	.36	.98	.98	<.001	.42	.005	.005	.20	.50
	***Clusters***										
		τ	−0.21	0.11	0.22	0.15	−0.30^e^	0.27	0.27	0.11	0.27
		*P* value	.22	.51	.20	.31	.04	.07	.07	.50	.13
	***Days of the week***										
		τ	−0.41^d^^,^^e^	0.14	−0.06	−0.02	−0.38^d^^,^^e^	0.01	0.01	−0.02	0.06
		*P* value	.02	.41	.71	.91	.01	.97	.97	.91	.75
	***Months of the year***										
		τ	0.3	0.05	0.14	−0.03	0.05	0.31^e^	0.31^e^	0.48^d^^,^^e^	0.2
		*P* value	.06	.77	.39	.85	.74	.04	.04	.002	.26
**E2 (higher IG is due to more COB)**											
	***Hours of the day***										
		τ	0.15	−0.05	0.14	0.08	0.19	0.35^e^	0.35^e^	0.17	0.18
		*P* value	.30	.75	.35	.55	.15	.01	.01	.21	.26
	***Clusters***										
		τ	−0.24	0.14	0.20	0.02	−0.35^e^	0.22	0.22	0.08	0.24
		*P* value	.16	.42	.25	.92	.02	.15	.15	.61	.19
	***Days of the week***										
		τ	−0.05	−0.1	0.07	−0.01	−0.02	−0.04	−0.04	0.02	−0.15
		*P* value	.76	.55	.66	.95	.88	.78	.78	.90	.38
	***Months of the year***										
		τ	0.35^e^	−0.05	0.16	−0.05	0.15	0.34^e^	0.34^e^	0.38^d^^,^^e^	0.13
		*P* value	.03	.77	.33	.73	.32	.03	.03	.02	.46
**U1 (more IOB is not due to more COB)**											
	***H*** ***ours of the day***										
		τ	0.42^d^^,^^e^	−0.31^e^	0.17	−0.13	0.36^d^^,^^e^	0.21	0.21	0.05	0.05
		*P* value	.004	.04	.24	.34	.007	.12	.12	.70	.75
	***Clusters***										
		τ	0.01	0.12	0.28	−0.03	0.02	0.28	0.28	0.25	0.12
		*P* value	.94	.46	.08	.86	.90	.05	.05	.10	.48
	***Days of the week***										
		τ	0.04	0.05	0.18	−0.21	0.01	0.16	0.16	0.36^d^^,^^e^	0.14
		*P* value	.80	.75	.27	.17	.96	.28	.28	.02	.42
	***Months of the year***										
		τ	0.23	−0.16	0.2	−0.09	−0.18	0.24	0.24	0.41^d^^,^^e^	0.46^d^^,^^e^
		*P* value	.16	.33	.21	.57	.23	.11	.11	.008	.008
**U2 (higher IG is not due to more COB)**											
	***Hours of the day***										
		τ	0.15	−0.05	0.14	0.08	0.19	0.35^e^	0.35^e^	0.17	0.18
		*P* value	.30	.75	.35	.55	.15	.01	.01	.21	.26
	***Clusters***										
		τ	0.14	−0.01	0.33^e^	0.00	−0.04	0.30^e^	0.30^e^	0.40^e^	0.19
		*P* value	.38	.98	.04	.98	.79	.04	.04	.01	.24
	***Days of the week***										
		τ	−0.05	−0.1	0.07	−0.01	−0.02	−0.04	−0.04	0.02	−0.15
		*P* value	.76	.55	.66	.95	.88	.78	.78	.90	.38
	***Months of the year***										
		τ	0.39^d^^,^^e^	−0.22	0.35^e^	0.07	0.15	0.34^e^	0.34^e^	0.41^d^^,^^e^	−0.02
		*P* value	.01	.18	.03	.61	.32	.02	.02	.008	.89
**U3 (more COB does not require more IOB)**											
	***Hours of the day***										
		τ	−0.03	0.21	−0.19	0.21	0.07	0.06	0.06	−0.02	−0.01
		*P* value	.85	.17	.19	.12	.61	.66	.66	.89	.93
	***Clusters***										
		τ	−0.16	0.19	−0.01	−0.04	−0.03	0.07	0.07	−0.11	0.08
		*P* value	.34	.29	.95	.78	.82	.64	.64	.48	.66
	***Days of the week***										
		τ	−0.06	0.22	−0.19	−0.02	−0.14	−0.2	−0.2	0.15	0.28
		*P* value	.74	.20	.26	.87	.36	.20	.20	.36	.12
	***Months of the year***										
		τ	0.18	−0.07	0.24	−0.07	0.12	0.41^d^^,^^e^	0.41^d^^,^^e^	0.45^d^^,^^e^	0.34^d^^,^^e^
		*P* value	.27	.66	.14	.66	.41	.006	.006	.003	.048

a COB: carbohydrates on board.

b IG: interstitial glucose.

c IOB: insulin on board.

d Power of ≥80%.

e Statistically significant association with *P*<.05 where we can reject the null hypothesis *τ*=0.

Our study achieved a power of 80% for associations with *τ*≥0.36. For associations with 0.3<τ<0.36, 2‐9 additional participants would be required. For *τ*≤0.3, we could not reject the null hypothesis that *τ*=0. A τ of 0.3 would require 41 participants, a τ of 0.24 would require 64 participants, a τ of 0.2 would require 91 participants, and a τ of 0.15 would require 163 participants.

### Comparison of IOB, COB, and IG

The comparison of IOB, COB, and IG across the 4 temporal dichotomies revealed varying results. For clusters of similar days, the Mann-Whitney *U* tests found significant differences for both IOB (*P*=.03, *d*=0.7) and IG (*P*=.02, *d*=0.67) but not COB (*P*=.08, *d*=0.55). For weekends versus workdays, no significant differences for IOB, COB, or IG were found (IOB *P*=.43, *d*=0.05; COB *P*=.45, *d*=0.15; IG *P*=.56, *d*=1.28). The same results were found for summer versus winter months (IOB *P*=.65, *d*=0.62; COB *P*=.19, *d*=1.09; IG *P*=.78, *d*=1.23) and year 1 versus year 2 (IOB *P*=.87, *d*=0.55; COB *P*=.53, *d*=0.48; IG *P*=.49, *d*=1.2). For all temporal dichotomies, the effect size *d* was larger for IG (0.67-1.28) than for IOB (0.43‐0.87) or COB (0.15‐0.55). Power analysis suggested that, for the clusters, we would need 27 (IOB), 30 (COB), and 44 (IG) participants to achieve a power of 80% (*α*=.05, 1-tailed). For the weekdays/weekends, we would need 13,696 (IOB), 1464 (COB), and 24 (IG). For the winter/summer months, we would need 88 (IOB), 30 (COB), and 26 (IG). Finally, for year 1/2, we would need 114 (IOB), 144 (COB), and 26 (IG). Therefore, most of these results are not conclusive for the number of participants included.

Grouping participants from these results by significant opposite differences highlighted the heterogeneity of the participants. Comparing the 2 clusters, 15 participants had lower IOB in cluster 1 (mean *d*=9.41, SD 5.47, range 3.56‐20.78), and 13 showed no significant differences. In addition, 13 participants had lower COB in cluster 1 (mean *d*=6.66, SD 2.99, range 3.1‐14.08), 3 had higher COB (mean *d*=5.07, SD 1.95, range 3.59‐7.27), and 12 showed no significant differences. A total of 15 participants had lower IG in cluster 1 (mean *d*=9.57, SD 5.06, range 3.23‐20.02), 2 had higher IG (mean *d*=3.75, SD 0.8, range 3.18‐4.31), and 11 showed no significant differences. Of the 15 people with lower IOB in cluster 1, one person had less IOB (difference in mean IOB of 0.86 U), while unexpectedly having significantly more COB (difference in mean COB of 5.33 grams), and their IG was also lower (difference in mean IG of 20.86 mg/dL) in cluster 1. Furthermore, 5 people had no significant differences in COB between clusters 1 and 2 despite having significantly lower IOB in 1 of them. Comparing weekdays and weekends revealed only 1 participant with lower IOB on weekends (*d*=3.27) and 1 individual with more IOB on weekends (*d*=4.21), while 26 showed no difference. No significant differences were observed for COB and IG. Comparing winter and summer months found 1 person with less IOB in summer (*d*=5.37), 2 individuals with more IOB in summer (mean *d*=7.78, SD 5.74, range 3.72‐11.84), and 5 people with no significant differences. No one had less COB in summer, 3 people had higher COB in summer (mean *d*=3.31, SD 0.28, range 3.12‐3.63), and for 5 people, there were no significant differences. Finally, no one had lower IG in summer, 2 participants had higher IG in summer (mean *d*=5.28, SD 2.49, range 3.52‐7.34), and for 6 people, there were no significant differences. Finally, comparing year 1 and 2 of AID use, we found that 5 participants had less IOB in year 2 (mean *d*=10.61, SD 5.58, range 4.35‐16.42), 2 people had more IOB (mean *d*=14.16, SD 1.54, range 13.07‐15.24), and for 9, there were no significant differences in IOB. For COB, 4 people had lower COB in year 2 (mean *d*=9.76, SD 3.59, range 6.36‐13.54), 3 people had higher COB (mean *d*=4.74, SD 1.39, range 3.62‐6.3), and for 9 people, there were no significant differences. For IG, 5 people had lower IG in year 2 (mean *d*=6.51, SD 2.22, range 3.87‐9.05), 4 individuals had higher IG (mean *d*=5.63, SD 1.31, range 3.71‐6.55), and 7 people had no significant differences.

**Figure 2.**
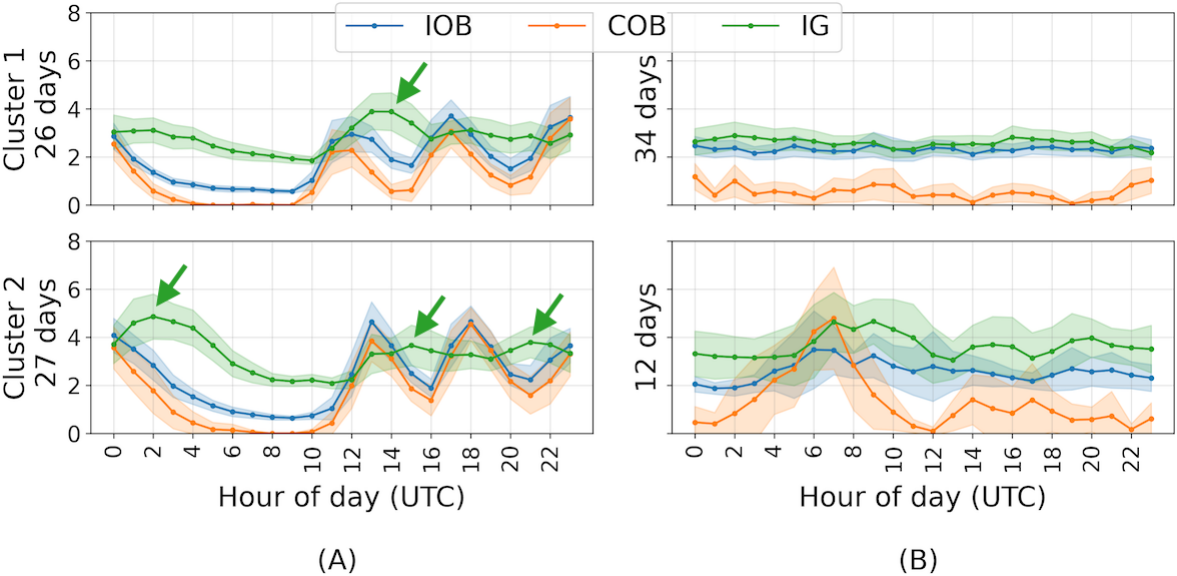
Cluster means (solid lines) and 95% CIs (bands) of (A) a participant with 3 meal spikes and an unexpected pattern of raised IG after meals and (B) another participant with flatter lines in cluster 1 compared to cluster 2. For (**A**), IOB matches COB more closely, while for (**B**), IOB matches IG more closely. COB: carbohydrates on board; IG: interstitial glucose; IOB: insulin on board.

Comparing the 2 clusters by visualizing the 95% CIs of mean IOB, COB, and IG for each cluster showed further interesting patterns, such as differences in the number of pronounced COB spikes, duration of hours with 0 COB, and days with flatter lines versus days with spikes. For 18 people, the number of pronounced COB spikes (presumably the main meals of the day) varied between the clusters (Figure 2B). Only 1 person had 3 COB spikes (presumably 3 big carbohydrate meals) in both clusters (Figure 2A), while 6 participants had no pronounced COB spikes in either cluster. Visually, COB varied more between the clusters (for 25 of the 28 people). This was perhaps due to COB forming distinct spikes, while IOB varied more frequently but subtly. For 24 people, the longest continuous hours of 0 COB were different between the 2 clusters. There were 18 people with 5 or more hours of 0 COB in at least 1 cluster, 8 in both, and 11 in neither.

**Figure 3.**
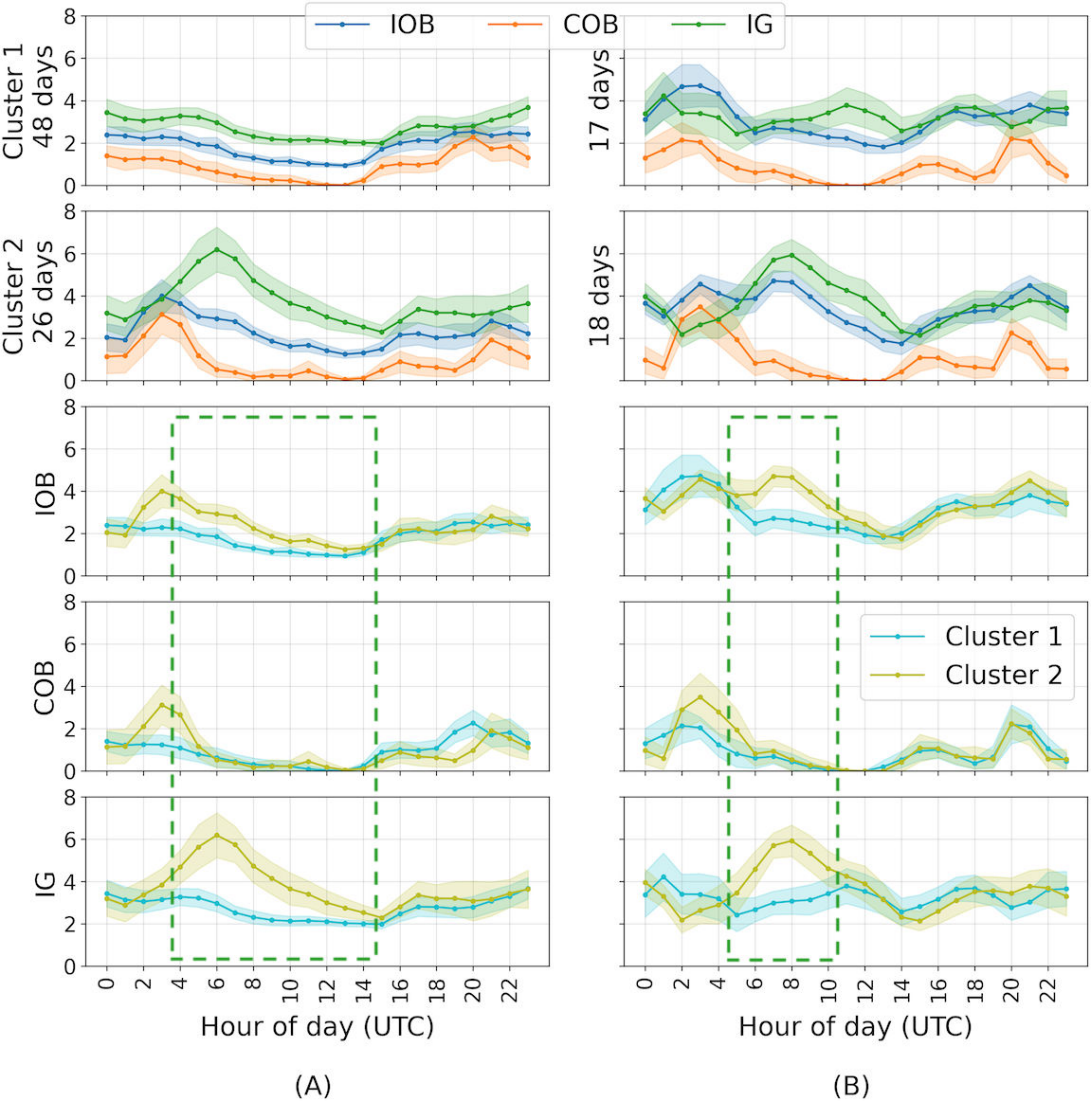
Cluster means (solid lines) and 95% CIs (bands) from 2 different participants (A) and (B) with a different “higher IG during the night despite significantly higher IOB” pattern. The first 2 rows show the 3 variates in cluster 1 and cluster 2. The bottom 3 rows show cluster 1 and 2 for each variate. COB: carbohydrates on board; IG: interstitial glucose; IOB: insulin on board.

In all, 12 participants had 1 cluster where IOB, COB, and IG varied more and another cluster with flatter lines. For 11 people, the cluster with flatter lines was more common (see Figure 2B for an example of this pattern). The most common unexpected pattern from visual inspections of the clustering results was a pattern where IG continues to rise after COB from meals has dropped (17 people; Figure 2A). Another common visually identifiable, unexpected pattern (11 people of 28) in the clustering results was higher IG during the night (see Figure 3 for 2 examples). This pattern occurred especially in the early part of the night (Figure 3A) and in some people through the night (Figure 3B). In addition, 10 participants with this pattern also had higher IOB but the AID’s correction was too small to avoid the higher IG. Finally, 3 people had higher IOB but not higher IG, and therefore the IOB correction matched their insulin needs well.

Looking into the similarities between the mean IOB, COB, and IG time series in the clusters, IOB and IG were most similar (mean distance 5.08, SD 2.25) and COB and IG were most different from each other (mean distance 11.43, SD 2.6). Interestingly, this changed for the first derivative of the means. In trend, IOB and COB were most similar (mean distance 1.48, SD 0.85) and COB and IG were still the most different from each other (mean distance 3.03, SD 1.51). Figure 2A shows an example of a person where IOB is visibly more like COB for both clusters, while Figure 2B shows another person where IOB and IG are visibly more like each other.

### Forecastability of IOB, COB, and IG

Testing which variate could forecast another revealed that at lag 1, IOB could forecast IG; COB could forecast IG; and IG could forecast COB most frequently. For 12 participants, this was true for both of their clusters; for another 12 participants, this was the case for 1 of their clusters; and for 4 participants, this was not the case. IG could forecast IOB least frequently (Table 6). A lag of 1 (meaning the value of variate 1 from 1 hour ago can be used to forecast the value of variate 2 now) gave the best result. For lags 2 and 3, fewer participants had Granger causalities between the variates. The mean derivative required to pass the ADF test for stationarity was 1.98 (SD 0.9, range 0‐3), indicating that momentum was most frequently stationary.

**Table 6.** The number of people for which one variate can forecast the other variate with a lag of 1 hour in both clusters, 1 cluster, or never, and the mean (SD) of the number of derivatives of insulin on board (IOB), carbohydrates on board (COB), and interstitial glucose (IG) required to achieve stationarity.

	Number of people			Mean (SD) derivative
	For both clusters	For 1 cluster	Never	
IOB forecasts COB	10	12	6	2.2 (0.91)
COB forecasts IOB	10	13	5	2.1 (0.9)
IOB forecasts IG	12	12	4	2.0 (0.87)
IG forecasts IOB	9	11	8	2.0 (0.9)
COB forecasts IG	12	12	4	1.97 (0.9)
IG forecasts COB	12	12	4	2.0 (0.94)

**Figure 4.**
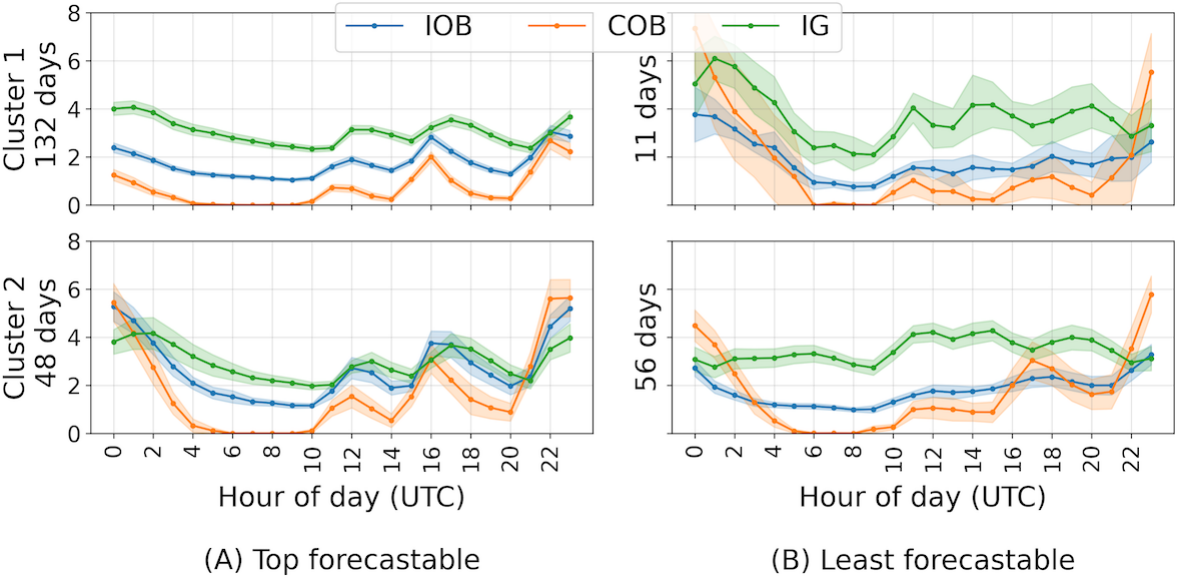
Cluster means (solid lines) and 95% CIs (bands) of (A) the participant with the top forecastable means and (B) the participant with the least forecastable means. COB: carbohydrates on board; IG: interstitial glucose; IOB: insulin on board.

Surprisingly, Granger causality changed for most people (21 of the 28) for 1 or more of the 2-pair permutations of IOB, COB, and IG between the 2 clusters. For 7 people, there was no difference in Granger causality between the 2 clusters. For 5 people, all 6 pair permutations of IOB, COB, and IG in both clusters differed. There were 7 people who had 1 cluster where IOB, COB, and IG could not be used to forecast each other at lag 1; for 4 of these, this was also true for lag 2; and for 3 of them, for lag 3. Figure 4 shows the cluster visualizations for the person for whom IOB, COB, and IG were best forecastable (Figure 4A) and the person for whom they were least forecastable (Figure 4B). For the best forecastable cluster means, IOB, COB, and IG could forecast each other in clusters 1 and 2 for lag 1 for all pairwise permutations. For cluster 1, this held for lag 2 and 3. For cluster 2, at lag 2 and 3, IG did not Granger cause IOB nor vice versa. For the least forecastable cluster means, in cluster 1, all IOB, COB, and IG permutations were not Granger causal for lag 1, 2, and 3. In cluster 2 COB, only Granger caused IOB at lag 1. No other Granger causalities were present for lag 1, 2, and 3.

## Discussion

### Principal Results

We discovered interesting temporal patterns in the insulin needs of people with T1D that cannot be explained by carbohydrate intake alone. Our study analyzed automatically recorded IOB, COB, and IG time series data from the AID systems of 29 participants. Compared to national T1D statistics, our participants had a lower HbA_1c_ (mean 46 mmol/mol) [67,68] and were early adopters [69] of insulin pumps (mean started in 2006), CGMs (mean started in 2014), and AIDs (mean started in 2017). Remarkably, unexpected patterns, such as when more insulin and higher glucose levels did not coincide with more carbohydrates, were as frequent as their expected counterparts. Overall, 9.9 participants (range 2.67‐21) had expected patterns and 13.5 (range 8‐23) had unexpected patterns (Table 3). Both expected and unexpected patterns were more frequent, and their effect sizes were larger (*d*>0.94), when comparing hours of the day and clusters of similar days compared to days of the week or months of the year (0.3<*d*<0.52). There was a considerable variety of patterns among the participants. For example, 3 participants did not have an expected pattern, while all participants had at least 1 unexpected pattern in at least 1 of the time resolutions. The number of observations and effect sizes of the patterns varied among the participants. A power of 80% was achieved for effect sizes around the median value and higher (Table 4).

Overall, the significant associations between demographic information and pattern frequency were of medium strength 0.31≤*τ*≤0.48 (*P*<.001 to .04; Table 5). Surprisingly, age, duration of T1D, average daily carbohydrates, basal insulin, and length of CGM and AID use were not significantly associated with the frequency of expected or unexpected patterns (0.01≤*τ*≤0.31). A higher HbA_1c_ increased the frequency of the expected pattern E2 (higher IG and higher COB) and unexpected patterns U1 (higher IOB but similar or lower COB) and U2 (higher IG but similar or lower COB), with 0.35≤*τ*≤0.42; in addition, higher HbA_1c_ decreased the frequency of expected pattern E1 (higher COB and higher IOB), with *τ*=−0.41. Participants who used an insulin pump for a shorter period had a higher frequency of the unexpected pattern U2 (higher IG but same or lower COB), with 0.33≤*τ*≤0.35. Lower mean IG was associated with an increased frequency of the expected patterns E1 (higher IOB and higher COB) and E2 (higher IG and higher COB), with 0.38≤*τ*≤0.3. Finally, higher mean IG was associated with an increase in the unexpected pattern U2 (higher IOB but same or lower COB), with *τ*=0.36. Note that these relationships did not hold for all the time resolutions. Similarly to the frequency of patterns, days of the week had the fewest significant associations. For the significant associations where *τ*≥0.36, the power was ≥80% at *α*=.05.

Mean IOB and IG significantly differed between the 2 clusters (IOB *P*=.03, *d*=0.7; IG *P*=.02, *d*=0.67; COB *P*=.08, *d*=0.55). No significant differences were found between workdays and weekends, winter and summer months, and the first and second year of AID data. However, visual analysis of the clustered days showed 2 common examples of unexpected patterns: a pattern with higher IG overnight (11 of 29 people) alongside higher IOB (10 of these 11 people; Figure 3) and a pattern where IG continued to rise after COB from meals dropped (17 of 29 people; Figure 2). In the first pattern, the AID system correctly raised IOB to deal with the increased IG but not sufficiently to avoid IG rising. Given the AID system had no information about the cause of this rise in IG (there is no higher COB during these times), it increased IOB cautiously. Further, we found that, measured in Euclidean distance between the means of each cluster, IOB and IG were most similar (mean distance 5.08, SD 2.25), and COB and IG were most different from each other (mean distance 11.43, SD 2.6). This demonstrates the AID system utilizing insulin to control IG by counteracting COB and unobserved confounders.

IOB, COB, and IG were all able to forecast each other for some of the participants in some of their clusters (Table 6). Which variable could forecast another and at what lag varied. This variation in the ability to predict IG from IOB and COB provides further evidence that unobserved confounding factors influence glucose regulation.

From a methods perspective, using a temporal view aided our discovery of patterns. This is perhaps not surprising given the long-lasting and often delayed effects of different factors on IG [35,72]. Although time series k-means clustering of the days uncovered many interesting patterns between IOB, COB, and IG, the clustering quality achieved was unsatisfactory (mean average silhouette score for Euclidean distance was 0.17, SD 0.09, range 0.06‐0.43). Our results suggest that, even after grouping the days into 2 clusters, many differences remained. This can be explained by people varying when and how often they eat, sleep, and do other activities that impact insulin needs. The improved silhouette scores for the SoftDTW distance measure further support this explanation.

### Comparison With Prior Work

Many studies research various factors that impact blood glucose regulation. Potential explanations for the nighttime high glucose pattern could be the impact of quality of sleep on glycemia [73] or the dawn phenomenon [74]. The second pattern of rising glucose levels after the carbohydrates have dropped could be due to the AID system’s method of calculating COB, which perhaps underestimates how long carbohydrates take to be fully absorbed either in general or for some meals where other nutritional components create a longer lasting or delayed rise of glucose. Again, there are studies available that suggest macronutrients [35] should be considered for insulin dosing. Another reason for unexpected patterns could be varying insulin absorption due to various factors such as malfunctioning infusion systems, lipohypertrophy, and temperature changes, which are being researched [72,75]. Like our study, previous studies were not able to consistently observe effects across all participants.

Various studies have examined the accurate prediction of blood glucose levels in T1D using different algorithms [7]. The researchers found that their algorithm works better on simulated patients. We conjecture that this is due to unexpected patterns not being simulated. Another study explored 12 learning algorithms and 13 feature sets to predict glucose levels [76]. They concluded that manually recorded diary data did not provide accurate predictions and suggested that CGM data might improve the situation. We found that whether COB and IOB can be used to forecast IG varies. Our findings suggest that, in many situations, more information than IG from CGM, IOB, and COB is required.

The participants studied seemed to be mindful about how much and when they ate carbohydrates. This was evident by the relatively low mean amount of carbohydrate intake reported (156.7 grams/day, SD 72.2, range 20‐330), as well as in the clustering of the days where 18 people had 1 cluster with lower carbohydrates (*P*<.001 to .02). Furthermore, 10 people reported eating a standard amount of carbohydrates per day (>130 grams/day), 17 people ate a low-carbohydrate diet (50‐130 grams/day), and 2 people ate a very low carbohydrate diet of <50 grams/day; measures for diet classification were obtained from [71]. Only 1 person reported eating more than 266 grams/day. Overall, the carbohydrates eaten were lower than the recommended amount of carbohydrates for the general population (267 grams/day for females, 333 grams/day for males, guidelines taken from [77]). Although carbohydrate counting and professional nutritional advice are part of the treatment guidelines for T1D, restricting carbohydrates is not recommended for people with T1D [78]. However, reducing the amount of carbohydrates eaten seems to be a successful glucose management strategy for many. A total of 12 people had flat lines of IG and IOB on days where COB was also a flat line, while IG varied more on days with COB spikes. Our results showed that the people we studied seemed to be cautious about their carbohydrate intake and their IG outcomes were more stable on days with fewer carbohydrate spikes. However, a “low” or “very low” carbohydrate diet remains a controversial T1D intervention with unclear long-term effects [2] and studies stress the importance of a professionally supported low-carbohydrate diet [66].

### Limitations

There are a few important limitations to consider. The OpenAPS Data Commons dataset used in this study might be biased due to circumstances that have led to participants donating their data: donation happens on a volunteer basis; the participants need access and funding for an insulin pump and continuous glucose monitor; and the participants need to feel competent and confident to navigate the process of setting up and running their open-source AID device. The participants in this study are early technology adopters and live in Western countries (the majority in North America). More research on different cohorts is required to understand if and how these characteristics have influenced the results.

Given the data are donated ad hoc, the amount of data varies between participants (the range of number of hours is 720‐9024; Table 2). We have included only participants who have at least 30 days of data, and we selected methods that can cope with comparing means from varying group sizes. However, limitations still exist. For some people, the data might be from consecutive days, while others have gaps. Further, some people have data from 19 different months and some from 3 different months; there are similar discrepancies for years (Table 2). Therefore, we cannot tell if the lower frequency of patterns in months of the year is due to not having at least 12 full months of data for everyone or because these patterns happen less frequently monthly. This would need to be investigated in a future study with more consecutive data for everyone. The significant relationships found between the frequency of patterns and the amount of data (Table 5) further support this.

Having only 29 participants limited some analyses. We could not find a significant difference in overall mean IOB, COB, and IG between workdays and weekends, winter and summer months, and first and second year of AID data. These results were inconclusive, particularly where our study did not achieve a power of 80% for the participants and effect sizes we observed. We have provided the number of participants required for a power of 80% to help plan future studies.

Furthermore, the OpenAPS AID device offers configurations that impact the IOB and COB calculations. On top of this, the software is regularly updated. We have not assessed the impact of settings or software changes in this study.

The time series k-means algorithm requires regularly sampled data with no gaps, which led us to create the hourly sampled daily time series. On one hand, aggregating multiple readings into a mean hourly reading lessens the impact of outliers, while on the other hand, it hides patterns that happened within the hour. Time series analysis methods generally expect regularly sampled and close to equal-length data. More work needs to be done on algorithms that can handle data that are irregularly sampled, with varying sampling intervals between variates, as well as missing data.

During preprocessing of the data, we decided to translate all timestamps to UTC to avoid jumps in time that are common for people who live in countries with multiple time zones like the United States and countries that follow daylight saving time. UTC also provides better anonymization of the data. However, it also means that the “hour of the day” is different for people depending on where they live. This means we cannot compare the same “hour of the day” from one person to another, as it might be nighttime for one participant and lunchtime for another.

Last, this study did not examine the differences in demographics between individuals who have a pattern and those who do not, nor did it research which confounding factors caused a pattern due to the dataset lacking high-frequency recordings of such factors. This would be interesting for future research and could help to stratify people with similar patterns and shed light on what leads to unexpected patterns. Note that for the 29 individuals selected in this study, there are only 2 female data donors. More data would be needed for such a study.

### Conclusions

In conclusion, our results show that changes in insulin needs due to factors beyond carbohydrate intake occur frequently. The AID device adjusts insulin in unexpected ways, which seems required for the narrow range of HbA_1c_ maintained (mean 46 mmol/mol, SD 7.9). This supports our hypothesis that factors beyond carbohydrates play a substantial role in euglycemia. For such factors to become more systematically included in clinical practice, we need to find a way to measure and utilize this information for insulin dosing decisions. This information could also help forecast IG, which we have shown is not consistently possible from IOB and COB alone. Our findings further demonstrate the heterogeneity of patterns in insulin needs among people with T1D and underline the need for personalized treatment approaches. Not only do people have different and often conflicting patterns, but some people also have no patterns. This increases the complexity of detecting such patterns and devising an approach for including them in insulin-dosing decisions. It also offers a potential explanation for why factors beyond carbohydrates are not yet systematically considered, measured, and quantified, and why adjusting insulin dosing for such factors is often left to the individual with T1D to decipher. We have not found characteristics that can predict which pattern people will follow, nor could we consistently relate their demographic information to their pattern frequency. Based on the relationships between pattern frequency and demographic information found, we can assume that HbA_1c_, the amount of carbohydrates eaten, and how long an insulin pump has been used impact the frequency of patterns. It remains to be seen if a cohort with less tech experience and higher HbA_1c_ would have similar patterns. Based on our findings, we would like to stress the importance of including a variety of participants when researching T1D and anticipating that methods that work for some cohorts might not work for others.

Until we have measurable and quantifiable information about factors that drive changes in insulin needs in unexpected ways, AID systems are left to adjust IOB cautiously, with the effect of IG going outside of euglycemia. More research is required to gain measurable and quantified information about these factors. This would be an enormous contribution to a better understanding of T1D and its treatment.

From a methods perspective, clustering days is helpful but crude. Future research could investigate segmenting and clustering time series dynamically based on changes in the relationships between IOB, COB, and IG. Our results show that patterns are more commonly found in finer time resolutions, which require methods that support irregularly sampled multivariate time series data, inherent to AID data and medical data in general.

Finally, to support future research, we would welcome long-term, open-access AID datasets that include a wide range of sensor measurements of possible factors and a diverse cohort of people with T1D. Such data would also aid research into the causalities behind these patterns.

## Supplementary material

10.2196/44384 Multimedia Appendix 1 Additional results.

## Abbreviations

ADF: augmented Dickey-Fuller
AID: automated insulin delivery
CGM: continuous glucose monitoring
COB: carbohydrates on board
DTW: dynamic time warping
HbA_1c_: glycated hemoglobin A_1c_
IG: interstitial glucose
IOB: insulin on board
T1D: type 1 diabetes

## References

[1] DiMeglio LA, Evans-Molina C, Oram RA (2018). Type 1 diabetes. Lancet.

[2] Holt RIG, DeVries JH, Hess-Fischl A (2021). The management of type 1 diabetes in adults. A consensus report by the American Diabetes Association (ADA) and the European Association for the Study of Diabetes (EASD). Diabetologia.

[3] Bertachi A, Ramkissoon CM, Bondia J, Vehí J (2018). Automated blood glucose control in type 1 diabetes: a review of progress and challenges. Endocrinol Diabet Nutr.

[4] Phillip M, Nimri R, Bergenstal RM (2023). Consensus recommendations for the use of automated insulin delivery technologies in clinical practice. Endocr Rev.

[5] Crabtree TSJ, McLay A, Wilmot EG (2019). DIY artificial pancreas systems: here to stay?. Pract Diab.

[6] Contreras I, Vehi J (2018). Artificial intelligence for diabetes management and decision support: literature review. J Med Internet Res.

[7] Munoz-Organero M (2020). Deep physiological model for blood glucose prediction in T1DM patients. Sensors (Basel).

[8] Jaloli M, Cescon M (2023). Long-term prediction of blood glucose levels in type 1 diabetes using a CNN-LSTM-based deep neural network. J Diabetes Sci Technol.

[9] San PP, Ling SH, Nguyen HT Deep learning framework for detection of hypoglycemic episodes in children with type 1 diabetes.

[10] Cescon M, DeSalvo DJ, Ly TT (2016). Early detection of infusion set failure during insulin pump therapy in type 1 diabetes. J Diabetes Sci Technol.

[11] Turksoy K, Roy A, Cinar A (2017). Real-time model-based fault detection of continuous glucose sensor measurements. IEEE Trans Biomed Eng.

[12] Kavakiotis I, Tsave O, Salifoglou A, Maglaveras N, Vlahavas I, Chouvarda I (2017). Machine learning and data mining methods in diabetes research. Comput Struct Biotechnol J.

[13] Hidalgo JI, Colmenar JM, Kronberger G, Winkler SM, Garnica O, Lanchares J (2017). Data based prediction of blood glucose concentrations using evolutionary methods. J Med Syst.

[14] Georga EI, Protopappas VC, Polyzos D, Fotiadis DI A predictive model of subcutaneous glucose concentration in type 1 diabetes based on random forests.

[15] Visentin R, Campos-Náñez E, Schiavon M (2018). The UVA/Padova type 1 diabetes simulator goes from single meal to single day. J Diabetes Sci Technol.

[16] Weisman A, Bai JW, Cardinez M, Kramer CK, Perkins BA (2017). Effect of artificial pancreas systems on glycaemic control in patients with type 1 diabetes: a systematic review and meta-analysis of outpatient randomised controlled trials. Lancet Diabetes Endocrinol.

[17] O’Donnell S, Lewis D, Marchante Fernández M (2019). Evidence on user-led innovation in diabetes technology (the OPEN project): protocol for a mixed methods study. JMIR Res Protoc.

[18] Knoll C, Peacock S, Wäldchen M (2022). Real‐world evidence on clinical outcomes of people with type 1 diabetes using open‐source and commercial automated insulin dosing systems: a systematic review. Diabet Med.

[19] Braune K, O’Donnell S, Cleal B (2019). Real-world use of do-it-yourself artificial pancreas systems in children and adolescents with type 1 diabetes: online survey and analysis of self-reported clinical outcomes. JMIR Mhealth Uhealth.

[20] Petruzelkova L, Soupal J, Plasova V (2018). Excellent glycemic control maintained by open-source hybrid closed-loop AndroidAPS during and after sustained physical activity. Diabetes Technol Ther.

[21] Lewis DM, Swain RS, Donner TW (2018). Improvements in A1C and time-in-range in DIY closed-loop (OpenAPS) users. Diabetes.

[22] Oliver N, Reddy M, Marriott C, Walker T, Heinemann L (2019). Open source automated insulin delivery: addressing the challenge. NPJ Digit Med.

[23] Shahid A, Lewis DM (2022). Large-scale data analysis for glucose variability outcomes with open-source automated insulin delivery systems. Nutrients.

[24] Khan FA, Zeb K, Al-Rakhami M, Derhab A, Bukhari SAC (2021). Detection and prediction of diabetes using data mining: a comprehensive review. IEEE Access.

[25] Lewis D #OpenAPS.org.

[26] #OpenAPS community Understanding the determine-basal logic — OpenAPS 000 documentation.

[27] #OpenAPS community Understanding Insulin on Board (IOB) Calculations — OpenAPS 000 documentation.

[28] Janež A, Guja C, Mitrakou A (2020). Insulin therapy in adults with type 1 diabetes mellitus: a narrative review. Diabetes Ther.

[29] Bell KJ, Barclay AW, Petocz P, Colagiuri S, Brand-Miller JC (2014). Efficacy of carbohydrate counting in type 1 diabetes: a systematic review and meta-analysis. Lancet Diabetes Endocrinol.

[30] McIntyre HD (2014). Dose adjustment for normal eating: a role for the expert patient?. Diabetes Metab J.

[31] Heller S, Lawton J, Amiel S (2014). Improving management of type 1 diabetes in the UK: the Dose Adjustment For Normal Eating (DAFNE) programme as a research test-bed. A mixed-method analysis of the barriers to and facilitators of successful diabetes self-management, a health economic analysis, a cluster randomised controlled trial of different models of delivery of an educational intervention and the potential of insulin pumps and additional educator input to improve outcomes. Prog Grants for Appl Res.

[32] Röder PV, Wu B, Liu Y, Han W (2016). Pancreatic regulation of glucose homeostasis. Exp Mol Med.

[33] Nakrani MN, Wineland RH, Physiology AF, Metabolism G (2023). Physiology, Glucose Metabolism.

[34] Liu L, Dattaroy D, Simpson KF (2021). Gq signaling in α cells is critical for maintaining euglycemia. JCI Insight.

[35] Evert AB (2020). Factors beyond carbohydrate to consider when determining meantime insulin doses: protein, fat, timing, and technology. Diabetes Spectr.

[36] Riddell MC, Peters AL (2023). Exercise in adults with type 1 diabetes mellitus. Nat Rev Endocrinol.

[37] Roep BO, Thomaidou S, van Tienhoven R, Zaldumbide A (2021). Type 1 diabetes mellitus as a disease of the β-cell (do not blame the immune system?). Nat Rev Endocrinol.

[38] Akl MG, Baccetto R, Stebbings BM, Li L, Widenmaier SB (2023). Euglycemia is affected by stress defense factor hepatocyte NRF1, but not NRF2. Biochem Biophys Res Commun.

[39] Toor S, Yardley JE, Momeni Z (2023). Type 1 diabetes and the menstrual cycle: where/how does exercise fit in?. Int J Environ Res Public Health.

[40] Beck RW, Bergenstal RM (2021). Beyond A1C-standardization of continuous glucose monitoring reporting: why it is needed and how it continues to evolve. Diabetes Spectr.

[41] Degen I (2022). Isabelladegen/insulin-need: find patterns in insulin need from the OpenAPS commons dataset. Code for NeurIPS TS4H workshop paper 2022. GitHub.

[42] (2023). Pandas-dev/pandas: pandas. Zenodo.

[43] Harris CR, Millman KJ, van der Walt SJ (2020). Array programming with NumPy. Nat New Biol.

[44] Virtanen P, Gommers R, Oliphant TE (2020). SciPy 1.0: fundamental algorithms for scientific computing in Python. Nat Methods.

[45] Asesh A, Biele C, Kacprzyk J, Kopeć W, Owsiński JW, Romanowski A, Sikorski M (2022). Digital Interaction and Machine Intelligence. MIDI’2021. Lecture Notes in Networks and Systems.

[46] Compare the effect of different scalers on data with outliers — scikit-learn 140 documentation. Scikit-learn.

[47] Rousseeuw PJ (1987). Silhouettes: a graphical aid to the interpretation and validation of cluster analysis. J Comput Appl Math.

[48] Vendramin L, Campello R, Hruschka ER (2010). Relative clustering validity criteria: a comparative overview. Stat Anal.

[49] Berndt DJ, Clifford J Using dynamic time warping to find patterns in time series. https://cdn.aaai.org/Workshops/1994/WS-94-03/WS94-03-031.pdf.

[50] Cuturi M, Blondel M Soft-DTW: a differentiable loss function for time-series.

[51] Degen I, Abdallah ZS (2022). Temporal patterns in insulin needs for type 1 diabetes. arXiv.

[52] Tavenard R, Faouzi J, Vandewiele G (2020). Tslearn, a machine learning toolkit for time series data. J Mach Learn Res.

[53] Pedregosa F, Michel V, Grisel O (2011). Scikit-learn: machine learning in Python. J Mach Learn Res.

[54] Campbell MJ (2021). Statistics at Square One.

[55] Kendall MG (1938). A new measure of rank correlation. Biometrika.

[56] Kendall MG, Gibbons JD (1990). Rank Correlation Methods.

[57] Arndt S, Turvey C, Andreasen NC (1999). Correlating and predicting psychiatric symptom ratings: Spearmans r versus Kendalls tau correlation. J Psychiatr Res.

[58] May JO, Looney SW (2020). Sample size charts for Spearman and Kendall coefficients. J Biom Biostat.

[59] Mann HB, Whitney DR (1947). On a test of whether one of two random variables is stochastically larger than the other. Ann Math Statist.

[60] Faul F, Erdfelder E, Lang AG, Buchner A (2007). G*Power 3: a flexible statistical power analysis program for the social, behavioral, and biomedical sciences. Behav Res Methods.

[61] Granger CWJ (1969). Investigating causal relations by econometric models and cross-spectral methods. Econometrica.

[62] Shojaie A, Fox EB (2022). Granger causality: a review and recent advances. Annu Rev Stat Appl.

[63] Dickey DA, Fuller WA (1979). Distribution of the estimators for autoregressive time series with a unit root. J Am Stat Assoc.

[64] Mushtaq R (2011). Augmented Dickey Fuller test. SSRN J.

[65] Seabold S, Perktold J Statsmodels: econometric and statistical modeling with Python.

[66] Turton JL, Brinkworth GD, Parker HM (2023). Effects of a low-carbohydrate diet in adults with type 1 diabetes management: a single arm non-randomised clinical trial. PLoS ONE.

[67] (2017). Type 1 diabetes in adults: diagnosis and management. National Institute for Health and Care Excellence.

[68] National Diabetes Audit 2021-22, Type 1 Diabetes - Overview. NHS Digital.

[69] Perez-Nieves M, Juneja R, Fan L, Meadows E, Lage MJ, Eby EL (2022). Trends in U.S. insulin use and glucose monitoring for people with diabetes: 2009-2018. J Diabetes Sci Technol.

[70] Lewis D, Leibrand S, #OpenAPS Community (2016). Real-world use of open source artificial pancreas systems. J Diabetes Sci Technol.

[71] Ozoran H, Matheou M, Dyson P, Karpe F, Tan GD (2023). Type 1 diabetes and low carbohydrate diets—Defining the degree of nutritional ketosis. Diabet Med.

[72] Gradel AKJ, Porsgaard T, Lykkesfeldt J (2018). Factors affecting the absorption of subcutaneously administered insulin: effect on variability. J Diabetes Res.

[73] Rechenberg K, Griggs S, Jeon S, Redeker N, Yaggi HK, Grey M (2020). Sleep and glycemia in youth with type 1 diabetes. J Pediatr Health Care.

[74] O’Neal TB, Luther EE (2023). StatPearls.

[75] Hauzenberger JR, Hipszer BR, Loeum C (2017). Detailed analysis of insulin absorption variability and the tissue response to continuous subcutaneous insulin infusion catheter implantation in swine. Diabetes Technol Ther.

[76] Borle NC, Ryan EA, Greiner R (2021). The challenge of predicting blood glucose concentration changes in patients with type I diabetes. Health Informatics J.

[77] Nutrition Science Team (2016). Government Dietary Recommendations for energy and nutrients for males and females aged 1-18 years and 19+ years. https://assets.publishing.service.gov.uk/media/5a749fece5274a44083b82d8/government_dietary_recommendations.pdf.

[78] Bolla AM, Caretto A, Laurenzi A, Scavini M, Piemonti L (2019). Low-carb and ketogenic diets in type 1 and type 2 diabetes. Nutrients.

[79] Lewis D Data commons. OpenAPS.org.

