# Supplementary material for: Beyond Expected Patterns in Insulin Needs of People With Type 1 Diabetes: Temporal Analysis of Automated Insulin Delivery Data
Source: JMIRx Med. 2024 Nov 27;5:e44384. doi: 10.2196/44384 (PMC11612581; doi:10.2196/44384)
Supplement: Multimedia Appendix 1 [file xmed-v5-e44384-s001.pdf]

Additional Results

Data and Population

Table S1: Distribution characteristics of insulin on board (IOB), carbohydrates on board (COB) and interstitial glucose (IG) for Group 1 (n=29) show non-normal distributions and wide ranges across the participants.

Measure and variates	mean (SD)	range
**Kurtosis**		
IOB	3.4 (2.73)	0.27-13.27
COB	8.07(6.25)	1.22-26.18
IG	1.98 (1.22)	-0.2-5.03
**Skew**		
IOB	1.53 (0.53)	0.68-3.28
COB	2.55 (0.89)	1.4-4.9
IG	1.17 (0.25)	0.66-1.76
**Number of modes**		
IOB	4.2 (5.4)	1-22
COB	1 (0)	N/A
IG	1.34 (0.61)	1-3

Clustering Similar Days

Table S2: Mean average silhouette score for the Euclidian, SoftDTW and DTW distance measures for k=2 and n=29 (Group 1). DTW stands for dynamic time warping.

Distance measure	mean (SD)	range
**Euclidean**	0.17 (0.09)	0.06-0.43
**SoftDTW**	0.32 (0.16)	0.04-0.76
**DTW**	0.18 (0.09)	0.03-0.43

Table S3: Number of clusters k, number of participants n for which this k is the best, average Euclidean silhouette score for k and n for which k is best and average Euclidean silhouette score for k=2 for the 'k best for n' participants.

k	k best for n	Avg. silhouette score for k (SD)	Avg. silhouette score for k=2 (SD)
**2**	21	0.17 (0.07)	0.17 (0.07)
**3**	1	0.14	0.13
**4**	1	0.08	0.07
**5**	3	0.12 (0.01)	0.094 (0.02)
**11**	1	0.07	0.05
**13**	1	0.19	0.13
**16**	1	0.08	0.07

Frequency of Expected and Unexpected Patterns

Table S4: Required observations n to achieve a power of 80% for the different time granularities (hours of the day, clusters, days of the week and months of the year) and various effect sizes d.

	Required observations n for a power of 80%			
**d**	**Hours of the day** α**=.0002**	**Clusters** α**=.0021**	**Days of the week** α**=.0024**	**Months of the year** α**=.0008**
**0.2**	1051	768	752	886
**0.3**	467	342	335	394
**0.5**	169	123	121	142
**0.8**	66	48	47	56
**1**	43	31	31	36
**1.5**	19	14	14	16
**2**	11	8	8	9

Relationships between Pattern Frequency and Factors

Table S5: Kendall's Tau associations between expected (E1-E3) and unexpected (U1-U3) patterns and the various factors for each time granularity. The highlighted cells have a statistically significant association with P<.05 where we can reject the null hypothesis t=0. The bold cells indicate a power of 80%. IOB stands for insulin on board, COB for carbohydrates on board and IG for interstitial glucose.

		**Age**	**Duration of T1D**	**Last A1C**	**Avg. Carbs**	**Avg. Insulin**	**Avg. Basal Insulin**	**Pumping since**	**CGM since**	**AID since**	**Mean IOB**	**Mean COB**	**Mean IG**	**Hours count**	**Days count**	**Months count**	**Years count**
n		25	26	25	25	23	24	25	25	25	29	29	29	29	29	29	29
n clusters		24	25	24	24	22	24	24	24	24	28	28	28	28	28	28	28
**E1 - Higher IOB is needed for higher COB & E3 - Higher COB needs higher IOB**																	
hours of the day	τ	-0.05	-0.09	0.13	0.25	0	-0.09	0	0.1	0.22	0.07	**0.45**	0.11	**0.38**	**0.38**	0.18	0.11
	P	.71	.51	.36	.08	.98	.55	.98	.49	.18	.59	**<.001**	.42	**.005**	**.005**	.20	.50
clusters	τ	0.13	0.03	-0.21	0.09	0.11	0.09	0.22	0.14	-0.11	0.12	0.15	-0.30	0.27	0.27	0.11	0.27
	P	.45	.86	.22	.58	.51	.58	.20	.41	.55	.44	.31	.04	.07	.07	.50	.13
days of the week	τ	0.28	0.3	**-0.41**	0.14	0.14	0.15	-0.06	-0.04	-0.03	0.03	-0.02	**-0.38**	0.01	0.01	-0.02	0.06
	P	.1	.07	**.02**	.40	.41	.38	.71	.80	.86	.86	.91	**.01**	.97	.97	.91	.75
months of the year	τ	-0.15	-0.11	0.3	-0.28	0.05	0.08	0.14	0.11	-0.14	0.27	-0.03	0.05	0.31	0.31	**0.48**	0.2
	P	0.35	0.48	0.06	0.09	0.77	0.64	0.39	0.51	0.44	0.07	0.85	0.74	.04	.04	**.002**	0.26
**E2 - Higher IG is due to higher COB**																	
hours of the day	τ	0.01	-0.15	0.15	0.24	-0.05	-0.06	0.14	0.2	0.14	0.05	0.08	0.19	0.35	0.35	0.17	0.18
	P	.96	.29	.30	.11	.75	.71	.35	.19	.37	.73	.55	.15	.01	.01	.21	.26
clusters	τ	0.13	0.14	-0.24	0.07	0.14	0.14	0.20	0.16	-0.17	0.07	0.02	-0.35	0.22	0.22	0.08	0.24
	P	.45	.39	.16	.69	.42	.41	.25	.35	.36	.65	.92	.02	.15	.15	.61	.19
days of the week	τ	-0.14	0.01	-0.05	0.04	-0.1	-0.09	0.07	-0.07	-0.04	0.18	-0.01	-0.02	-0.04	-0.04	0.02	-0.15

	P	.39	.96	.76	.80	.55	.56	.66	.66	.81	.23	.95	.88	.78	.78	.90	.38
months of the year	τ	-0.18	-0.09	0.35	-0.14	-0.05	0.01	0.16	0.07	-0.12	0.23	-0.05	0.15	0.34	0.34	**0.38**	0.13
	P	.26	.57	.03	.40	.77	.94	.33	.68	.50	.13	.73	.32	.03	.03	**.02**	.46
**U1 - Higher IOB is not due to higher COB**																	
hours of the day	τ	-0.08	-0.16	**0.42**	0.10	-0.31	-0.22	0.17	0.13	-0.01	-0.26	-0.13	**0.36**	0.21	0.21	0.05	0.05
	P	.59	.26	**.004**	.50	.04	.13	.24	.40	.94	.05	.34	**.007**	0.12	0.12	.70	.75
clusters	τ	-0.10	-0.07	0.01	0.20	0.12	-0.05	0.28	0.25	-0.01	-0.03	-0.03	0.02	0.28	0.28	0.25	0.12
	P	.51	.65	.94	.22	.46	.73	.08	.13	.96	.83	.86	.90	.05	.05	.10	.48
days of the week	τ	-0.15	0.07	0.04	-0.16	0.05	0.16	0.18	-0.01	-0.11	0.1	-0.21	0.01	0.16	0.16	**0.36**	0.14
	P	.35	.66	.80	.34	.75	.34	.27	.93	.55	.49	.17	0.96	.28	.28	**.02**	.42
months of the year	τ	-0.15	-0.13	0.23	-0.22	-0.16	0.04	0.2	0.14	-0.14	0.09	-0.09	-0.18	0.24	0.24	**0.41**	**0.46**
	P	.34	.43	.16	.18	.33	.80	.21	.39	.44	.53	.57	.23	.11	.11	**.008**	**.008**
**U2 - Higher IG is not due to higher COB**																	
hours of the day	τ	0.01	-0.15	0.15	0.24	-0.05	-0.06	0.14	0.2	0.14	0.05	0.08	0.19	0.35	0.35	0.17	0.18
	P	.96	.29	.30	.11	.75	.71	.35	.19	.37	.73	.55	.15	0.01	0.01	.21	.26
clusters	τ	-0.19	-0.18	0.14	-0.03	-0.01	-0.12	0.33	0.30	0.05	-0.06	0.00	-0.04	0.30	0.30	0.40	0.19
	P	.22	.25	.38	.84	.98	.46	.04	.06	.78	.69	.98	.79	.04	.04	.01	.24
days of the week	τ	-0.14	0.01	-0.05	0.04	-0.1	-0.09	0.07	-0.07	-0.04	0.18	-0.01	-0.02	-0.04	-0.04	0.02	-0.15
	P	.39	.96	.76	.80	.55	.56	.66	.66	.81	.23	.95	.88	.78	.78	.90	.38
months of the year	τ	-0.31	-0.3	**0.39**	-0.18	-0.22	-0.18	0.35	0.14	-0.20	0.01	0.07	0.15	0.34	0.34	**0.41**	-0.02
	P	.05	.06	**.01**	.25	.18	.27	.03	.40	.26	.92	.61	.32	.02	.02	**.008**	.89
**U3 - Higher COB does not require higher IOB**																	
hours of the day	τ	-0.09	0.13	-0.03	0.2	0.21	0.1	-0.19	0.06	0.22	0.11	0.21	0.07	0.06	0.06	-0.02	-0.01
	P	.52	.37	.85	.18	.17	.50	.19	.67	.18	.41	.12	.61	.66	.66	.89	.93
clusters	τ	0.23	0.21	-0.16	0.14	0.19	0.04	-0.01	-0.09	-0.28	-0.06	-0.04	-0.03	0.07	0.07	-0.11	0.08
	P	.18	.21	.34	.41	.29	.79	.95	.60	.14	.70	.78	.82	.64	.64	.48	.66
days of the week	τ	-0.01	-0.05	-0.06	-0.26	0.22	0.11	-0.19	-0.12	-0.03	0.08	-0.02	-0.14	-0.2	-0.2	0.15	0.28
	P	.93	.75	.74	.13	.20	.51	.26	.48	.87	.59	.87	.36	.20	.20	.36	.12
months of the year	τ	-0.04	-0.24	0.18	-0.08	-0.07	0.02	0.24	0.07	-0.14	0.07	-0.07	0.12	**0.41**	**0.41**	**0.45**	0.34
	P	.81	.12	.27	.60	.66	.91	.14	.67	.44	.63	.66	.41	**.006**	**.006**	**.003**	.048
